# Expanding the Galaxy’s reference data

**DOI:** 10.1093/bioadv/vbac030

**Published:** 2022-04-29

**Authors:** Nagampalli VijayKrishna, Jayadev Joshi, Nate Coraor, Jennifer Hillman-Jackson, Dave Bouvier, Marius van den Beek, Ignacio Eguinoa, Frederik Coppens, John Davis, Michał Stolarczyk, Nathan C Sheffield, Simon Gladman, Gianmauro Cuccuru, Björn Grüning, Nicola Soranzo, Helena Rasche, Bradley W Langhorst, Matthias Bernt, Dan Fornika, David Anderson de Lima Morais, Michel Barrette, Peter van Heusden, Mauro Petrillo, Antonio Puertas-Gallardo, Alex Patak, Hans-Rudolf Hotz, Daniel Blankenberg

**Affiliations:** 1 Genomic Medicine Institute, Cleveland Clinic, Cleveland, OH 44195, USA; 2 Department of Biochemistry and Molecular Biology, Penn State University, University Park, PA 16802, USA; 3 VIB Center for Plant Systems Biology, 9052 Ghent, Belgium; 4 Department of Plant Biotechnology and Bioinformatics, Ghent University, 9052 Ghent, Belgium; 5 Department of Biology, Johns Hopkins University, Baltimore, MD 21218, USA; 6 Center for Public Health Genomics, University of Virginia, Charlottesville, VA 22903, USA; 7 University of Melbourne, Melbourne, VIC, Australia; 8 University of Freiburg, Freiburg im Breisgau, Germany; 9 Earlham Institute, Norwich Research Park, Norwich, UK; 10 Clinical Bioinformatics Group, Department of Pathology, Erasmus Medical Center, 3015 CN Rotterdam, The Netherlands; 11 New England Biolabs, Ipswich, MA 01938, USA; 12 Department Computational Biology, Helmholtz Centre for Environmental Research, UFZ, 04318 Leipzig, Germany; 13 BC Centre for Disease Control Public Health Laboratory, Vancouver, BC, Canada; 14 Centre de Calcul Scientifique, Université de Sherbrooke, Sherbrooke, QC, Canada; 15 South African Medical Research Council Bioinformatics Unit, South African National Bioinformatics Institute, University of the Western Cape, Bellville, South Africa; 16 European Commission, Joint Research Centre (JRC), Ispra, Italy; 17 Friedrich Miescher Institute for Biomedical Research, Basel, Switzerland; 18 SIB Swiss Institute of Bioinformatics, Basel, Switzerland; 19 Department of Molecular Medicine, Cleveland Clinic Lerner College of Medicine, Case Western Reserve University, Cleveland, OH 44195, USA

## Abstract

**Summary:**

Properly and effectively managing reference datasets is an important task for many bioinformatics analyses. Refgenie is a reference asset management system that allows users to easily organize, retrieve and share such datasets. Here, we describe the integration of refgenie into the Galaxy platform. Server administrators are able to configure Galaxy to make use of reference datasets made available on a refgenie instance. In addition, a Galaxy Data Manager tool has been developed to provide a graphical interface to refgenie’s remote reference retrieval functionality. A large collection of reference datasets has also been made available using the CVMFS (CernVM File System) repository from GalaxyProject.org, with mirrors across the USA, Canada, Europe and Australia, enabling easy use outside of Galaxy.

**Availability and implementation:**

The ability of Galaxy to use refgenie assets was added to the core Galaxy framework in version 22.01, which is available from https://github.com/galaxyproject/galaxy under the Academic Free License version 3.0. The refgenie Data Manager tool can be installed via the Galaxy ToolShed, with source code managed at https://github.com/BlankenbergLab/galaxy-tools-blankenberg/tree/main/data_managers/data_manager_refgenie_pull and released using an MIT license. Access to existing data is also available through CVMFS, with instructions at https://galaxyproject.org/admin/reference-data-repo/. No new data were generated or analyzed in support of this research.

## 1 Introduction

Among the primary resources required when performing biomedical genomic analyses are collections of reference datasets and annotations. These include sequences and features, such as genes, locations of epigenetic modifications, regulatory regions, etc. as well as derived datasets such as index files. Popular examples of derived datasets include FASTA sequence indexes created with SAMtools (Li *et al.*, 2009) and sequencing-read mapping tools such as Bowtie 2 (Langmead and Salzberg, 2012) or BWA (Li and Durbin, 2009). While generating these derived data is often relatively straightforward to accomplish, important attention to detail is required to ensure compatibility across analysis and analysis environments. For example, running the command ‘bwa index -a bwtsw reference.fa’ will create a set of index files on the provided reference sequence with the BWT-SW algorithm that can then be used by the BWA align function on user provided sequencing reads. However, in order to allow for reproducible analyses, not only do genome build versions of the reference sequences (e.g. hg19 versus hg38) and tool versions (e.g. BWA version 0.7.17-r1188) need to be recorded, but also algorithmic choices (e.g. algorithm for constructing BWT index: ‘bwtsw’ or ‘is’). Moreover, from a resource management perspective, it would be ideal for all users of a computing platform to share a single set of references, as opposed to each individual user creating and using their own copy on a filesystem. This has led to the development of several software solutions for reference genome resource management.

We have previously developed the Galaxy Data Manager framework (Blankenberg *et al.*, 2014b) which enables provenance-backed and reproducible reference datasets inside of Galaxy (Giardine *et al.*, 2005; Jalili *et al.*, 2020). Galaxy is an open-source platform for accessible, reproducible and transparent computational research. It provides a user-friendly, integrative analysis and visualization environment that ensures reproducibility and accessibility to tools, disparate datasets, heterogeneous compute resources, and is backed by community supported training materials. In addition to the ability to deploy Galaxy on one’s own hardware or within the Cloud, free public servers are available at https://usegalaxy.org, https://usegalaxy.eu and elsewhere. The Data Manager framework leverages the Galaxy tool system to enable community-driven creation and dissemination of reference dataset build recipes as Galaxy tools. These Data Manager tools can be installed on-demand from the ToolShed (Blankenberg *et al.*, 2014a) to allow Galaxy administrators to fetch, build and install new reference data. All underlying tool dependencies [SAMtools, BWA, STAR (Dobin *et al.*, 2013), Kraken (Wood and Salzberg, 2014), etc.] are well defined with versions and can be automatically and reproducibly resolved using e.g. Bioconda (Grüning *et al.*, 2018) and Docker. As with any Galaxy tool, multiple versions of a Data Manager can be installed and executed, with provenance and reproducibility maintained. Data Manager tools can be accessed using the Galaxy graphical user interface, a representational state transfer application programming interface [REST [representational state transfer]ful application programming interface (API)] or by using command-line scripts. Currently, over 70 Data Manager tools have been created and shared on the ToolShed by the Galaxy community.

Refgenie (Stolarczyk *et al.*, 2020) has been recently published as a reference genome resource manager. Refgenie provides a command-line interface to download, build and access reference genome assembly assets (i.e. reference sequences, mapping indexes, etc.). It can also be controlled via an API from other software. There are currently 24 recipes available for building assets locally. Refgenie can also provide a lightweight web server enabling sharing of local resources.

In light of its potential to become a widely accepted standard for reference data management, we have enabled the use of refgenie-based assets inside of Galaxy. Users are able to seamlessly access refgenie-defined references within Galaxy tools, and administrators are able to access refgenie’s ability to download reference datasets from remote resources. This approach enables the simultaneous creation and use of reference files by both Data Managers and refgenie within Galaxy.

Moreover, the adoption of refgenie by the Galaxy community could make high quality reference datasets and derived assets available for the broad scientific community. Data Managers, with over 250 assets available from the main Galaxy servers, have been shown to provide provenance-backed creation of reference datasets within a Galaxy instance, but were not originally designed for distribution or reuse outside of that Galaxy server. However, refgenie was designed as a standalone cross-platform reference management system. The adoption of a standalone reference management platform external to Galaxy has the potential to enhance the availability of reference data to the wider scientific community, similarly to the benefits experienced when Galaxy’s tool dependency framework was deprecated in favor of Bioconda.

## 2 Methods

We have extended the Galaxy Data Manager framework to interoperate with refgenie, a standalone reference genome manager.

### 2.1 Using refgenie assets in Galaxy

Integration of refgenie with Galaxy is available as of Galaxy version 22.01. Enabling refgenie usage within Galaxy is accomplished by following an optional two-step process: installing refgenie and configuring Galaxy.

A server administrator must first install refgenie using the standard prescribed instructions and take note of the path selected for its genome configuration file (see also https://galaxyproject.org/admin/refgenie/).

**Fig. 1.**
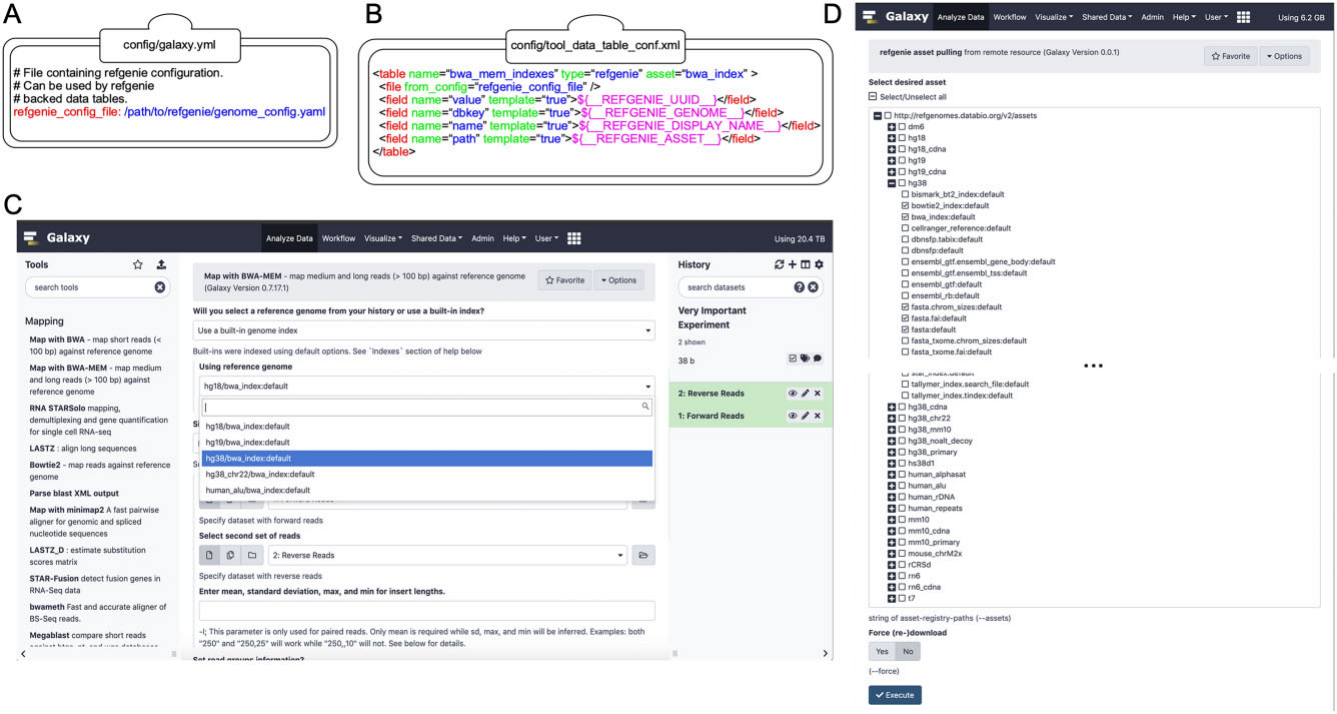
Extending Galaxy’s reference data with refgenie. (**A**) Setting the value of ‘refgenie_config_file’ to the previously chosen genome configuration file path within the primary Galaxy configuration file (e.g. ‘galaxy.yml’). (**B**) Example data table mapping between refgenie assets and Galaxy data tables for the BWA tool. Cheetah templating language is used to specify mappings between values, with several pre-populated refgenie variables available as shown. (**C**) refgenie assets are available for users to select and use in the Galaxy BWA tool. In this example, the user is mapping a set of paired-end sequencing reads against the hg38 genome. (**D**) A dynamically generated list of available remote refgenie assets are listed for an administrator to select in the ‘refgenie pull’ Galaxy Data Manager tool

Second, refgenie is enabled in Galaxy by setting the ‘refgenie_config_file’ value to the previously chosen genome configuration file path within the primary Galaxy configuration file (e.g. ‘galaxy.yml’; Fig. 1A). This setting enables the default mappings between refgenie asset names and values into Galaxy data table entries. Currently, default mappings have been defined for genome build identifiers, chromosome sizes and sequences, SAMtools FASTA indexes, Bowtie 2 indexes, BWA indexes and HISAT2 indexes. Additional mappings can be defined as shown in Figure 1B. The assets loaded from refgenie, are merged with those from Galaxy’s native Data Managers. This approach provides maximum flexibility for the administrator while creating a single unified access point from the Galaxy user’s perspective. In fact, it is possible for an administrator to define multiple different refgenie configurations within a single Galaxy instance, making the assets defined in each resource available as a combined reference collection in Galaxy. This can be helpful, for example, if the administrator wants to provide access to the prebuilt collection of datasets in CVMFS (CernVM File System) (see below) along with additional locally maintained refgenie datasets.

Once the refgenie integration has been configured, the users of the Galaxy server will be able to select the refgenie-provided datasets in Galaxy tools that make use of the data tables for which a mapping is defined (Fig. 1C).

### 2.2 Retrieving additional refgenie assets in Galaxy

An initial refgenie installation contains no data. However, the administrator is able to search, download and install assets into refgenie from public remote servers. Any asset loaded into refgenie using the standard approaches will be made available within Galaxy. To facilitate management of the assets, a Galaxy Data Manager tool has been created to provide a graphical interface to refgenie, which is now available in the Galaxy ToolShed (Blankenberg *et al.*, 2014a). When a Galaxy administrator accesses this Data Manager, a list of available remote assets is dynamically populated and displayed (Fig. 1D). These available references are organized by remote resource, genome build, asset type and tag. The administrator selects one or more assets to retrieve, and then clicks ‘Execute’. An external process is queued, using Galaxy’s job management system, which calls refgenie’s standard command-line tools to perform the requested actions. Once refgenie has completed, Galaxy loads the new values and makes them available to users. This Data Manager can also be controlled using the standard Galaxy tool API.

### 2.3 A CVMFS mirror

The refgenie software provides reference asset downloads from http://refgenomes.databio.org/. To increase reliability and availability, we have created a mirror of all of Galaxy and refgenie reference data available in a CernVM-FS (Blomer *et al.*, 2011) (CVMFS) repository. CVMFS is a caching, HTTP-based filesystem with a Filesystem in Userspace (FUSE) (mount) client. The Galaxy CVMFS repository distributes the data in multiple replicas across the USA, Canada, Europe and Australia. Moreover, it is possible to make the mirrored data directly available on any other server (see https://galaxyproject.org/admin/reference-data-repo/). When initially mounted, CVMFS does not consume any additional local disk space. Instead, as files are accessed, they are pulled from one of the replica (Stratum 1) servers to a local disk-based cache of a configurable size. This allows the administrator to directly load the refgenie configuration from e.g. /cvmfs/refgenomes-databio.galaxyproject.org/genomes_config.yaml, without needing to predetermine which assets the users may desire.

The presented approach of data distribution allows access to the same reference data through Galaxy as well as e.g. command line, enabling reproducible analysis beyond the Galaxy community. Additional optimizations, e.g. using HTTP proxies or alien cache, are easily deployable across data centers, HPC clusters and local labs, greatly facilitating scalability.

## 3 Conclusions

We have extended the Galaxy platform to support the use of externally managed reference datasets using refgenie. Galaxy is able to seamlessly merge refgenie-provided references with those specified within its native Data Manager framework. We have provided a Data Manager tool that enables Galaxy administrators to view and pull additional refgenie assets graphically or using an API. This allows administrators to easily populate a Galaxy instance with standardized references to built-in data, improving interoperability of analysis among the now widely extended network of public Galaxy servers (https://galaxyproject.org/use/). Galaxy administrators are able to use standard Data Managers to build additional reference artifacts based upon refgenie provided asset sources. While these reference datasets will be available to Galaxy users, there is not yet a way to push them back into refgenie. Furthermore, although refgenie contains its own system for building assets, due to the lack of several important features, such as a system for declaring dependency requirements, adding this functionality to the Data Manager framework is left as a future endeavor.

We are pleased to report that this integration between refgenie and Galaxy was achieved without the need for architectural modifications to either tool and hope to see other examples of such collaboration in the future. The availability of these reference datasets via CVMFS, refgenie, Galaxy Data Managers and other standard web protocols provides a globally accessible network of provenance-backed reference datasets that are useful, accessible and interoperable to a diverse range of researchers.

## Ethics approval and consent to participate

Not applicable.

## Consent for publication

Not applicable.
